# Supplementary material for: Overlapping cell population expression profiling and regulatory inference in C. elegans
Source: BMC Genomics. 2016 Feb 29;17:159. doi: 10.1186/s12864-016-2482-z (PMC4772325; doi:10.1186/s12864-016-2482-z)
Supplement: Additional file 13: — Web supplement. (DOC 21 kb) [file 12864_2016_2482_MOESM13_ESM.zip › sortWeb/clusters/hier.300.clusters/67.html]

Cluster 67 

## Cluster 67

### Expression

| cnd-1 rep. 1 | cnd-1 rep. 2 | cnd-1 rep. 3 | pha-4 rep. 1 | pha-4 rep. 2 | pha-4 rep. 3 | ceh-27 | ceh-36 | ceh-6 | F21D5.9 | mir-57 | mls-2 | pal-1 | pros-1 | ttx-3 | unc-130 | hlh-16 | irx-1 | ceh-6 (+) hlh-16 (+) | ceh-6 (+) hlh-16 (-) | ceh-6 (-) hlh-16 (+) | cnd-1 singlets | pha-4 singlets | 0 | 60 | 120 | 150 | 180 | 240 | 330 | 390 | 420 | 480 | 540 | 570 | 600 | 630 | 660 | NAME | Functional description |
| --- | --- | --- | --- | --- | --- | --- | --- | --- | --- | --- | --- | --- | --- | --- | --- | --- | --- | --- | --- | --- | --- | --- | --- | --- | --- | --- | --- | --- | --- | --- | --- | --- | --- | --- | --- | --- | --- | --- | --- |
|  |  |  |  |  |  |  |  |  |  |  |  |  |  |  |  |  |  |  |  |  |  |  |  |  |  |  |  |  |  |  |  |  |  |  |  |  |  | F08D12.12 |  |
|  |  |  |  |  |  |  |  |  |  |  |  |  |  |  |  |  |  |  |  |  |  |  |  |  |  |  |  |  |  |  |  |  |  |  |  |  |  | K09C6.10 |  |
|  |  |  |  |  |  |  |  |  |  |  |  |  |  |  |  |  |  |  |  |  |  |  |  |  |  |  |  |  |  |  |  |  |  |  |  |  |  | C24A8.10 |  |
|  |  |  |  |  |  |  |  |  |  |  |  |  |  |  |  |  |  |  |  |  |  |  |  |  |  |  |  |  |  |  |  |  |  |  |  |  |  | T10A3.4 |  |
|  |  |  |  |  |  |  |  |  |  |  |  |  |  |  |  |  |  |  |  |  |  |  |  |  |  |  |  |  |  |  |  |  |  |  |  |  |  | *acr-24* | AcetylCholine Receptor |
|  |  |  |  |  |  |  |  |  |  |  |  |  |  |  |  |  |  |  |  |  |  |  |  |  |  |  |  |  |  |  |  |  |  |  |  |  |  | *npr-28* | NeuroPeptide Receptor family |
|  |  |  |  |  |  |  |  |  |  |  |  |  |  |  |  |  |  |  |  |  |  |  |  |  |  |  |  |  |  |  |  |  |  |  |  |  |  | *srz-20* | Serpentine Receptor, class Z |
|  |  |  |  |  |  |  |  |  |  |  |  |  |  |  |  |  |  |  |  |  |  |  |  |  |  |  |  |  |  |  |  |  |  |  |  |  |  | F13B12.6 |  |
|  |  |  |  |  |  |  |  |  |  |  |  |  |  |  |  |  |  |  |  |  |  |  |  |  |  |  |  |  |  |  |  |  |  |  |  |  |  | Y52E8A.3 |  |
|  |  |  |  |  |  |  |  |  |  |  |  |  |  |  |  |  |  |  |  |  |  |  |  |  |  |  |  |  |  |  |  |  |  |  |  |  |  | *lite-1* | high-energy LIghT unrEsponsive |
|  |  |  |  |  |  |  |  |  |  |  |  |  |  |  |  |  |  |  |  |  |  |  |  |  |  |  |  |  |  |  |  |  |  |  |  |  |  | M28.4 |  |
|  |  |  |  |  |  |  |  |  |  |  |  |  |  |  |  |  |  |  |  |  |  |  |  |  |  |  |  |  |  |  |  |  |  |  |  |  |  | Y62H9A.4 |  |
|  |  |  |  |  |  |  |  |  |  |  |  |  |  |  |  |  |  |  |  |  |  |  |  |  |  |  |  |  |  |  |  |  |  |  |  |  |  | *twk-39* | TWiK family of potassium channels |
|  |  |  |  |  |  |  |  |  |  |  |  |  |  |  |  |  |  |  |  |  |  |  |  |  |  |  |  |  |  |  |  |  |  |  |  |  |  | *twk-17* | TWiK family of potassium channels |
|  |  |  |  |  |  |  |  |  |  |  |  |  |  |  |  |  |  |  |  |  |  |  |  |  |  |  |  |  |  |  |  |  |  |  |  |  |  | Y71G12B.31 |  |
|  |  |  |  |  |  |  |  |  |  |  |  |  |  |  |  |  |  |  |  |  |  |  |  |  |  |  |  |  |  |  |  |  |  |  |  |  |  | *ocr-3* | Osm-9 and Capsaicin receptor-Related |
|  |  |  |  |  |  |  |  |  |  |  |  |  |  |  |  |  |  |  |  |  |  |  |  |  |  |  |  |  |  |  |  |  |  |  |  |  |  | C24A11.12 |  |
|  |  |  |  |  |  |  |  |  |  |  |  |  |  |  |  |  |  |  |  |  |  |  |  |  |  |  |  |  |  |  |  |  |  |  |  |  |  | E02C12.12 |  |
|  |  |  |  |  |  |  |  |  |  |  |  |  |  |  |  |  |  |  |  |  |  |  |  |  |  |  |  |  |  |  |  |  |  |  |  |  |  | *sup-17* | SUPpressor |
|  |  |  |  |  |  |  |  |  |  |  |  |  |  |  |  |  |  |  |  |  |  |  |  |  |  |  |  |  |  |  |  |  |  |  |  |  |  | F26F12.3 |  |
|  |  |  |  |  |  |  |  |  |  |  |  |  |  |  |  |  |  |  |  |  |  |  |  |  |  |  |  |  |  |  |  |  |  |  |  |  |  | *ptr-21* | PaTched Related family |
|  |  |  |  |  |  |  |  |  |  |  |  |  |  |  |  |  |  |  |  |  |  |  |  |  |  |  |  |  |  |  |  |  |  |  |  |  |  | 2RSSE.3 |  |
|  |  |  |  |  |  |  |  |  |  |  |  |  |  |  |  |  |  |  |  |  |  |  |  |  |  |  |  |  |  |  |  |  |  |  |  |  |  | *pll-1* | PhosphoLipase C Like |
|  |  |  |  |  |  |  |  |  |  |  |  |  |  |  |  |  |  |  |  |  |  |  |  |  |  |  |  |  |  |  |  |  |  |  |  |  |  | C54G6.3 |  |
|  |  |  |  |  |  |  |  |  |  |  |  |  |  |  |  |  |  |  |  |  |  |  |  |  |  |  |  |  |  |  |  |  |  |  |  |  |  | *osm-9* | OSMotic avoidance abnormal |
|  |  |  |  |  |  |  |  |  |  |  |  |  |  |  |  |  |  |  |  |  |  |  |  |  |  |  |  |  |  |  |  |  |  |  |  |  |  | *gcy-15* | Guanylyl CYclase |
|  |  |  |  |  |  |  |  |  |  |  |  |  |  |  |  |  |  |  |  |  |  |  |  |  |  |  |  |  |  |  |  |  |  |  |  |  |  | *acr-5* | AcetylCholine Receptor |
|  |  |  |  |  |  |  |  |  |  |  |  |  |  |  |  |  |  |  |  |  |  |  |  |  |  |  |  |  |  |  |  |  |  |  |  |  |  | *lgc-39* | Ligand-Gated ion Channel |
|  |  |  |  |  |  |  |  |  |  |  |  |  |  |  |  |  |  |  |  |  |  |  |  |  |  |  |  |  |  |  |  |  |  |  |  |  |  | *dur-1* | Dauer Up-Regulated |
|  |  |  |  |  |  |  |  |  |  |  |  |  |  |  |  |  |  |  |  |  |  |  |  |  |  |  |  |  |  |  |  |  |  |  |  |  |  | *pept-2* | PEPTide transporter family |
|  |  |  |  |  |  |  |  |  |  |  |  |  |  |  |  |  |  |  |  |  |  |  |  |  |  |  |  |  |  |  |  |  |  |  |  |  |  | *trp-4* | TRP (transient receptor potential) channel family |
|  |  |  |  |  |  |  |  |  |  |  |  |  |  |  |  |  |  |  |  |  |  |  |  |  |  |  |  |  |  |  |  |  |  |  |  |  |  | H40L08.3 |  |
|  |  |  |  |  |  |  |  |  |  |  |  |  |  |  |  |  |  |  |  |  |  |  |  |  |  |  |  |  |  |  |  |  |  |  |  |  |  | ZK688.1 |  |
|  |  |  |  |  |  |  |  |  |  |  |  |  |  |  |  |  |  |  |  |  |  |  |  |  |  |  |  |  |  |  |  |  |  |  |  |  |  | E04F6.6 |  |
|  |  |  |  |  |  |  |  |  |  |  |  |  |  |  |  |  |  |  |  |  |  |  |  |  |  |  |  |  |  |  |  |  |  |  |  |  |  | B0238.12 |  |
|  |  |  |  |  |  |  |  |  |  |  |  |  |  |  |  |  |  |  |  |  |  |  |  |  |  |  |  |  |  |  |  |  |  |  |  |  |  | F58D5.6 |  |
|  |  |  |  |  |  |  |  |  |  |  |  |  |  |  |  |  |  |  |  |  |  |  |  |  |  |  |  |  |  |  |  |  |  |  |  |  |  | *spe-12* | defective SPErmatogenesis |
|  |  |  |  |  |  |  |  |  |  |  |  |  |  |  |  |  |  |  |  |  |  |  |  |  |  |  |  |  |  |  |  |  |  |  |  |  |  | *srj-40* | Serpentine Receptor, class J |
|  |  |  |  |  |  |  |  |  |  |  |  |  |  |  |  |  |  |  |  |  |  |  |  |  |  |  |  |  |  |  |  |  |  |  |  |  |  | C54G4.3 |  |
|  |  |  |  |  |  |  |  |  |  |  |  |  |  |  |  |  |  |  |  |  |  |  |  |  |  |  |  |  |  |  |  |  |  |  |  |  |  | ZK353.4 |  |
|  |  |  |  |  |  |  |  |  |  |  |  |  |  |  |  |  |  |  |  |  |  |  |  |  |  |  |  |  |  |  |  |  |  |  |  |  |  | D2007.2 |  |
|  |  |  |  |  |  |  |  |  |  |  |  |  |  |  |  |  |  |  |  |  |  |  |  |  |  |  |  |  |  |  |  |  |  |  |  |  |  | T07D1.3 |  |
|  |  |  |  |  |  |  |  |  |  |  |  |  |  |  |  |  |  |  |  |  |  |  |  |  |  |  |  |  |  |  |  |  |  |  |  |  |  | *scl-9* | SCP-Like extracellular protein |
|  |  |  |  |  |  |  |  |  |  |  |  |  |  |  |  |  |  |  |  |  |  |  |  |  |  |  |  |  |  |  |  |  |  |  |  |  |  | *ttll-9* | Tubulin Tyrosine Ligase Like |
|  |  |  |  |  |  |  |  |  |  |  |  |  |  |  |  |  |  |  |  |  |  |  |  |  |  |  |  |  |  |  |  |  |  |  |  |  |  | F53A2.11 |  |
|  |  |  |  |  |  |  |  |  |  |  |  |  |  |  |  |  |  |  |  |  |  |  |  |  |  |  |  |  |  |  |  |  |  |  |  |  |  | *sru-13* | Serpentine Receptor, class U |
|  |  |  |  |  |  |  |  |  |  |  |  |  |  |  |  |  |  |  |  |  |  |  |  |  |  |  |  |  |  |  |  |  |  |  |  |  |  | *rig-6* | neuRonal IGCAM |
|  |  |  |  |  |  |  |  |  |  |  |  |  |  |  |  |  |  |  |  |  |  |  |  |  |  |  |  |  |  |  |  |  |  |  |  |  |  | F53A2.1 |  |
|  |  |  |  |  |  |  |  |  |  |  |  |  |  |  |  |  |  |  |  |  |  |  |  |  |  |  |  |  |  |  |  |  |  |  |  |  |  | *str-164* | Seven TM Receptor |
|  |  |  |  |  |  |  |  |  |  |  |  |  |  |  |  |  |  |  |  |  |  |  |  |  |  |  |  |  |  |  |  |  |  |  |  |  |  | *str-4* | Seven TM Receptor |
|  |  |  |  |  |  |  |  |  |  |  |  |  |  |  |  |  |  |  |  |  |  |  |  |  |  |  |  |  |  |  |  |  |  |  |  |  |  | *ptr-9* | PaTched Related family |
|  |  |  |  |  |  |  |  |  |  |  |  |  |  |  |  |  |  |  |  |  |  |  |  |  |  |  |  |  |  |  |  |  |  |  |  |  |  | *oac-56* | O-ACyltransferase homolog |
|  |  |  |  |  |  |  |  |  |  |  |  |  |  |  |  |  |  |  |  |  |  |  |  |  |  |  |  |  |  |  |  |  |  |  |  |  |  | F56H9.6 |  |
|  |  |  |  |  |  |  |  |  |  |  |  |  |  |  |  |  |  |  |  |  |  |  |  |  |  |  |  |  |  |  |  |  |  |  |  |  |  | *col-89* | COLlagen |
|  |  |  |  |  |  |  |  |  |  |  |  |  |  |  |  |  |  |  |  |  |  |  |  |  |  |  |  |  |  |  |  |  |  |  |  |  |  | C16B8.2 |  |
|  |  |  |  |  |  |  |  |  |  |  |  |  |  |  |  |  |  |  |  |  |  |  |  |  |  |  |  |  |  |  |  |  |  |  |  |  |  | *ugt-59* | UDP-GlucuronosylTransferase |
|  |  |  |  |  |  |  |  |  |  |  |  |  |  |  |  |  |  |  |  |  |  |  |  |  |  |  |  |  |  |  |  |  |  |  |  |  |  | ZK455.5 |  |
|  |  |  |  |  |  |  |  |  |  |  |  |  |  |  |  |  |  |  |  |  |  |  |  |  |  |  |  |  |  |  |  |  |  |  |  |  |  | M01A12.3 |  |
|  |  |  |  |  |  |  |  |  |  |  |  |  |  |  |  |  |  |  |  |  |  |  |  |  |  |  |  |  |  |  |  |  |  |  |  |  |  | *col-53* | COLlagen |
|  |  |  |  |  |  |  |  |  |  |  |  |  |  |  |  |  |  |  |  |  |  |  |  |  |  |  |  |  |  |  |  |  |  |  |  |  |  | *cutl-4* | CUTiclin-Like |
|  |  |  |  |  |  |  |  |  |  |  |  |  |  |  |  |  |  |  |  |  |  |  |  |  |  |  |  |  |  |  |  |  |  |  |  |  |  | *col-174* | COLlagen |
|  |  |  |  |  |  |  |  |  |  |  |  |  |  |  |  |  |  |  |  |  |  |  |  |  |  |  |  |  |  |  |  |  |  |  |  |  |  | W05G11.4 |  |
|  |  |  |  |  |  |  |  |  |  |  |  |  |  |  |  |  |  |  |  |  |  |  |  |  |  |  |  |  |  |  |  |  |  |  |  |  |  | *mam-5* | MAM (Meprin, A5-protein, PTPmu) domain protein |
|  |  |  |  |  |  |  |  |  |  |  |  |  |  |  |  |  |  |  |  |  |  |  |  |  |  |  |  |  |  |  |  |  |  |  |  |  |  | ZC443.1 |  |
|  |  |  |  |  |  |  |  |  |  |  |  |  |  |  |  |  |  |  |  |  |  |  |  |  |  |  |  |  |  |  |  |  |  |  |  |  |  | *col-154* | COLlagen |
|  |  |  |  |  |  |  |  |  |  |  |  |  |  |  |  |  |  |  |  |  |  |  |  |  |  |  |  |  |  |  |  |  |  |  |  |  |  | *col-186* | COLlagen |
|  |  |  |  |  |  |  |  |  |  |  |  |  |  |  |  |  |  |  |  |  |  |  |  |  |  |  |  |  |  |  |  |  |  |  |  |  |  | F38E11.13 |  |
|  |  |  |  |  |  |  |  |  |  |  |  |  |  |  |  |  |  |  |  |  |  |  |  |  |  |  |  |  |  |  |  |  |  |  |  |  |  | *cutl-17* | CUTiclin-Like |
|  |  |  |  |  |  |  |  |  |  |  |  |  |  |  |  |  |  |  |  |  |  |  |  |  |  |  |  |  |  |  |  |  |  |  |  |  |  | C36B7.5 |  |
|  |  |  |  |  |  |  |  |  |  |  |  |  |  |  |  |  |  |  |  |  |  |  |  |  |  |  |  |  |  |  |  |  |  |  |  |  |  | *col-33* | COLlagen |
|  |  |  |  |  |  |  |  |  |  |  |  |  |  |  |  |  |  |  |  |  |  |  |  |  |  |  |  |  |  |  |  |  |  |  |  |  |  | *cutl-8* | CUTiclin-Like |
|  |  |  |  |  |  |  |  |  |  |  |  |  |  |  |  |  |  |  |  |  |  |  |  |  |  |  |  |  |  |  |  |  |  |  |  |  |  | *ptr-17* | PaTched Related family |
|  |  |  |  |  |  |  |  |  |  |  |  |  |  |  |  |  |  |  |  |  |  |  |  |  |  |  |  |  |  |  |  |  |  |  |  |  |  | ZK1010.4 |  |
|  |  |  |  |  |  |  |  |  |  |  |  |  |  |  |  |  |  |  |  |  |  |  |  |  |  |  |  |  |  |  |  |  |  |  |  |  |  | R07B1.6 |  |
|  |  |  |  |  |  |  |  |  |  |  |  |  |  |  |  |  |  |  |  |  |  |  |  |  |  |  |  |  |  |  |  |  |  |  |  |  |  | F53B2.10 |  |
|  |  |  |  |  |  |  |  |  |  |  |  |  |  |  |  |  |  |  |  |  |  |  |  |  |  |  |  |  |  |  |  |  |  |  |  |  |  | T01G9.1 |  |
|  |  |  |  |  |  |  |  |  |  |  |  |  |  |  |  |  |  |  |  |  |  |  |  |  |  |  |  |  |  |  |  |  |  |  |  |  |  | *grl-18* | GRound-Like (grd related) |
|  |  |  |  |  |  |  |  |  |  |  |  |  |  |  |  |  |  |  |  |  |  |  |  |  |  |  |  |  |  |  |  |  |  |  |  |  |  | *col-177* | COLlagen |
|  |  |  |  |  |  |  |  |  |  |  |  |  |  |  |  |  |  |  |  |  |  |  |  |  |  |  |  |  |  |  |  |  |  |  |  |  |  | F46F5.1 |  |
|  |  |  |  |  |  |  |  |  |  |  |  |  |  |  |  |  |  |  |  |  |  |  |  |  |  |  |  |  |  |  |  |  |  |  |  |  |  | *clec-246* | C-type LECtin |
|  |  |  |  |  |  |  |  |  |  |  |  |  |  |  |  |  |  |  |  |  |  |  |  |  |  |  |  |  |  |  |  |  |  |  |  |  |  | *cyp-33C6* | CYtochrome P450 family |
|  |  |  |  |  |  |  |  |  |  |  |  |  |  |  |  |  |  |  |  |  |  |  |  |  |  |  |  |  |  |  |  |  |  |  |  |  |  | 2RSSE.4 |  |
|  |  |  |  |  |  |  |  |  |  |  |  |  |  |  |  |  |  |  |  |  |  |  |  |  |  |  |  |  |  |  |  |  |  |  |  |  |  | C42D8.11 |  |
|  |  |  |  |  |  |  |  |  |  |  |  |  |  |  |  |  |  |  |  |  |  |  |  |  |  |  |  |  |  |  |  |  |  |  |  |  |  | *ace-3* | abnormal AcetylCholinEsterase |
|  |  |  |  |  |  |  |  |  |  |  |  |  |  |  |  |  |  |  |  |  |  |  |  |  |  |  |  |  |  |  |  |  |  |  |  |  |  | *hot-7* | Homolog of Odr-2 (Two) |
|  |  |  |  |  |  |  |  |  |  |  |  |  |  |  |  |  |  |  |  |  |  |  |  |  |  |  |  |  |  |  |  |  |  |  |  |  |  | *snf-3* | Sodium: Neurotransmitter symporter Family |
|  |  |  |  |  |  |  |  |  |  |  |  |  |  |  |  |  |  |  |  |  |  |  |  |  |  |  |  |  |  |  |  |  |  |  |  |  |  | *lst-2* | Lateral Signaling Target |
|  |  |  |  |  |  |  |  |  |  |  |  |  |  |  |  |  |  |  |  |  |  |  |  |  |  |  |  |  |  |  |  |  |  |  |  |  |  | K04C2.7 |  |
|  |  |  |  |  |  |  |  |  |  |  |  |  |  |  |  |  |  |  |  |  |  |  |  |  |  |  |  |  |  |  |  |  |  |  |  |  |  | *nhr-214* | Nuclear Hormone Receptor family |
|  |  |  |  |  |  |  |  |  |  |  |  |  |  |  |  |  |  |  |  |  |  |  |  |  |  |  |  |  |  |  |  |  |  |  |  |  |  | C17G10.6 |  |
|  |  |  |  |  |  |  |  |  |  |  |  |  |  |  |  |  |  |  |  |  |  |  |  |  |  |  |  |  |  |  |  |  |  |  |  |  |  | *cutl-21* | CUTiclin-Like |
|  |  |  |  |  |  |  |  |  |  |  |  |  |  |  |  |  |  |  |  |  |  |  |  |  |  |  |  |  |  |  |  |  |  |  |  |  |  | *mam-8* | MAM (Meprin, A5-protein, PTPmu) domain protein |
|  |  |  |  |  |  |  |  |  |  |  |  |  |  |  |  |  |  |  |  |  |  |  |  |  |  |  |  |  |  |  |  |  |  |  |  |  |  | F43C1.1 |  |
|  |  |  |  |  |  |  |  |  |  |  |  |  |  |  |  |  |  |  |  |  |  |  |  |  |  |  |  |  |  |  |  |  |  |  |  |  |  | *col-88* | COLlagen |
|  |  |  |  |  |  |  |  |  |  |  |  |  |  |  |  |  |  |  |  |  |  |  |  |  |  |  |  |  |  |  |  |  |  |  |  |  |  | Y45F10D.14 |  |
|  |  |  |  |  |  |  |  |  |  |  |  |  |  |  |  |  |  |  |  |  |  |  |  |  |  |  |  |  |  |  |  |  |  |  |  |  |  | Y54F10AM.12 |  |
|  |  |  |  |  |  |  |  |  |  |  |  |  |  |  |  |  |  |  |  |  |  |  |  |  |  |  |  |  |  |  |  |  |  |  |  |  |  | F07A11.13 |  |
|  |  |  |  |  |  |  |  |  |  |  |  |  |  |  |  |  |  |  |  |  |  |  |  |  |  |  |  |  |  |  |  |  |  |  |  |  |  | F36D4.t2 |  |
|  |  |  |  |  |  |  |  |  |  |  |  |  |  |  |  |  |  |  |  |  |  |  |  |  |  |  |  |  |  |  |  |  |  |  |  |  |  | W01B6.6 |  |
|  |  |  |  |  |  |  |  |  |  |  |  |  |  |  |  |  |  |  |  |  |  |  |  |  |  |  |  |  |  |  |  |  |  |  |  |  |  | T04C12.7 |  |
|  |  |  |  |  |  |  |  |  |  |  |  |  |  |  |  |  |  |  |  |  |  |  |  |  |  |  |  |  |  |  |  |  |  |  |  |  |  | *nhr-231* | Nuclear Hormone Receptor family |
|  |  |  |  |  |  |  |  |  |  |  |  |  |  |  |  |  |  |  |  |  |  |  |  |  |  |  |  |  |  |  |  |  |  |  |  |  |  | F35E8.6 |  |
|  |  |  |  |  |  |  |  |  |  |  |  |  |  |  |  |  |  |  |  |  |  |  |  |  |  |  |  |  |  |  |  |  |  |  |  |  |  | W07B8.4 |  |
|  |  |  |  |  |  |  |  |  |  |  |  |  |  |  |  |  |  |  |  |  |  |  |  |  |  |  |  |  |  |  |  |  |  |  |  |  |  | R153.3 |  |
|  |  |  |  |  |  |  |  |  |  |  |  |  |  |  |  |  |  |  |  |  |  |  |  |  |  |  |  |  |  |  |  |  |  |  |  |  |  | F34D10.8 |  |
|  |  |  |  |  |  |  |  |  |  |  |  |  |  |  |  |  |  |  |  |  |  |  |  |  |  |  |  |  |  |  |  |  |  |  |  |  |  | C30B5.6 |  |
|  |  |  |  |  |  |  |  |  |  |  |  |  |  |  |  |  |  |  |  |  |  |  |  |  |  |  |  |  |  |  |  |  |  |  |  |  |  | *dhhc-5* | DHHC-types zinc finger protein |
|  |  |  |  |  |  |  |  |  |  |  |  |  |  |  |  |  |  |  |  |  |  |  |  |  |  |  |  |  |  |  |  |  |  |  |  |  |  | C15B12.8 |  |
|  |  |  |  |  |  |  |  |  |  |  |  |  |  |  |  |  |  |  |  |  |  |  |  |  |  |  |  |  |  |  |  |  |  |  |  |  |  | *gba-4* | beta-GlucocereBrosidAse |
|  |  |  |  |  |  |  |  |  |  |  |  |  |  |  |  |  |  |  |  |  |  |  |  |  |  |  |  |  |  |  |  |  |  |  |  |  |  | *srh-10* | Serpentine Receptor, class H |
|  |  |  |  |  |  |  |  |  |  |  |  |  |  |  |  |  |  |  |  |  |  |  |  |  |  |  |  |  |  |  |  |  |  |  |  |  |  | ZK546.19 |  |
|  |  |  |  |  |  |  |  |  |  |  |  |  |  |  |  |  |  |  |  |  |  |  |  |  |  |  |  |  |  |  |  |  |  |  |  |  |  | F15A4.6 |  |

### Phenotypes enriched

none found

### Anatomy terms enriched

none found

### GO terms enriched

|  |  |  |
| --- | --- | --- |
| **GO term** | **Number of genes** | **FDR-corrected p-value** |
| membrane | 19 | 0.0016 |
| integral component of membrane | 16 | 0.0092 |
| channel activity | 8 | 0.0120 |
| substrate-specific transmembrane transporter activity | 9 | 0.0450 |

### Expression clusters enriched

none found

### Motifs enriched

|  |  |  |  |  |  |
| --- | --- | --- | --- | --- | --- |
| **Motif** | **Logo** | **Possible orthologs** | **Number of motifs in cluster** | **Enrichment** | **FDR corrected p** |
| Otp\_3496 |  | alr-1 (0.67) ceh-18 ceh-43 npax-3 ceh-1 lin-39 ceh-12 ceh-45 ceh-53 | 47 | 2.42 | 5.5e-07 |
| Msx3\_3206 |  | alr-1 (0.67) ceh-10 ceh-1 lin-39 eyg-1 ceh-45 | 47 | 2.36 | 1.2e-06 |
| V$CART1\_01 |  | alr-1 (0.67) ceh-43 lin-39 ceh-12 | 46 | 2.37 | 1.6e-06 |
| pTH5673 |  | alr-1 (0.67) ceh-10 lim-7 ceh-23 ceh-2 ceh-18 ceh-43 ceh-30 ceh-14 ceh-1 dsc-1 ceh-31 lim-6 egl-5 cog-1 mls-2 pal-1 ceh-16 lin-39 lim-4 and 9 others  [full list] | 41 | 2.45 | 6.4e-06 |
| Nkx1-2\_3214 |  | ceh-30 | 41 | 2.44 | 7.2e-06 |
| Hoxd1\_3448 |  | ceh-12 | 45 | 2.28 | 7.9e-06 |
| Dlx1\_1741 |  | ceh-43 | 43 | 2.29 | 1.6e-05 |
| Nkx1-1\_3856 |  | ceh-30 | 39 | 2.44 | 1.7e-05 |
| ELF2\_f1 |  | lin-1 C24A1.2 | 68 | 1.70 | 2.5e-05 |
| pTH5539 |  | unc-120 | 19 | 4.34 | 2.6e-05 |
| Elf3\_3876 |  | C24A1.2 | 59 | 1.84 | 2.8e-05 |
| Hlxb9\_3422 |  | ceh-12 | 40 | 2.32 | 3.5e-05 |
| pTH6569 |  | ceh-43 | 40 | 2.30 | 4.2e-05 |
| RAX\_1 |  | alr-1 (0.67) lim-7 ceh-43 ceh-1 ceh-31 ceh-9 ceh-16 lin-39 | 46 | 2.07 | 6.5e-05 |
| Emx2\_3420 |  | ceh-2 | 41 | 2.22 | 7.2e-05 |
| V$AREB6\_04 |  | ztf-6 gei-11 C34D1.1 | 53 | 1.89 | 8.6e-05 |
| pTH3120 |  | che-1 K11D2.4 tbp-1 | 57 | 1.80 | 1.1e-04 |
| Hmx1\_3423 |  | ceh-9 | 48 | 1.97 | 1.2e-04 |
| DLX2\_f1 |  | hmbx-1 lin-31 ceh-43 lin-39 Y116A8C.22 | 39 | 2.22 | 1.5e-04 |
| MA0169.1 |  | ceh-8 (0.76) alr-1 (0.67) lim-7 ceh-19 ceh-24 ceh-30 ceh-43 ceh-1 ceh-31 ceh-9 cog-1 lin-39 | 47 | 1.98 | 1.6e-04 |
| Vax1\_3499 |  | C02F12.10 | 42 | 2.10 | 1.8e-04 |
| pTH6562 |  | ceh-5 | 42 | 2.09 | 1.9e-04 |
| SOX2\_6 |  | sox-4 grh-1 | 44 | 2.04 | 2.0e-04 |
| ETV2\_1 |  | lin-1 C24A1.2 | 53 | 1.83 | 2.1e-04 |
| V$S8\_01 |  | ceh-45 | 71 | 1.56 | 2.4e-04 |
| pTH6436 |  | ceh-53 | 41 | 2.10 | 2.4e-04 |
| Vax2\_3500 |  | C02F12.10 | 42 | 2.07 | 2.4e-04 |
| Ftz\_Cell\_FBgn0001077 |  | lim-7 lin-39 | 45 | 1.99 | 2.6e-04 |
| Pax7\_3783 |  | alr-1 (0.67) lim-7 ceh-18 | 46 | 1.95 | 3.1e-04 |
| Dlx2\_2273 |  | ceh-43 | 39 | 2.14 | 3.2e-04 |
| Evx1\_3952 |  | ceh-53 | 40 | 2.11 | 3.2e-04 |
| pTH9380 |  | mel-28 | 88 | 1.38 | 3.4e-04 |
| Dlx3\_1030 |  | ceh-43 | 42 | 2.04 | 3.4e-04 |
| V$OCT1\_06 |  | ceh-18 ztf-9 | 58 | 1.71 | 3.5e-04 |
| pTH10796 |  | F10B5.3 (0.63) hsf-1 | 69 | 1.56 | 4.0e-04 |
| En1\_3123 |  | ceh-16 ceh-53 | 43 | 2.00 | 4.0e-04 |
| Gsh2\_3990 |  | ceh-31 | 39 | 2.10 | 4.8e-04 |
| V$YY1\_01 |  | lsy-2 | 62 | 1.63 | 5.4e-04 |
| Hoxa3\_2783 |  | lin-39 | 24 | 2.83 | 5.8e-04 |
| Blimp-1\_SANGER\_5\_FBgn0035625 |  | blmp-1 (0.52) | 82 | 1.41 | 6.0e-04 |
| pTH9149 |  | ztf-30 | 38 | 2.11 | 6.0e-04 |
| Bsx\_3483 |  | ceh-31 | 40 | 2.04 | 6.2e-04 |
| Tbp\_pr781 |  | tbp-1 | 78 | 1.44 | 8.1e-04 |
| pTH2283 |  | odd-2 | 69 | 1.53 | 8.2e-04 |
| Lmx1a\_2238 |  | lim-7 lim-6 ceh-16 | 38 | 2.07 | 8.4e-04 |
| pTH6478 |  | lim-7 | 40 | 2.01 | 8.5e-04 |
| GM12878\_PBX3\_HudsonAlpha |  | lin-31 dro-1 ceh-20 nfya-2 | 25 | 2.68 | 8.6e-04 |
| Hoxb6\_3428 |  | lin-39 | 40 | 2.01 | 8.8e-04 |
| pTH6449 |  | ceh-43 | 37 | 2.09 | 9.1e-04 |
| FOXC1\_1 |  | lin-31 let-381 | 81 | 1.40 | 1.0e-03 |
| pTH4325 |  | ceh-18 | 67 | 1.54 | 1.0e-03 |
| FOXJ3\_f2 |  | lin-31 let-381 daf-16 fkh-7 | 63 | 1.58 | 1.1e-03 |
| pTH6268 |  | ceh-2 | 22 | 2.81 | 1.5e-03 |
| MA0037.2 |  | elt-1 | 43 | 1.88 | 1.6e-03 |
| pTH6445 |  | ceh-5 | 50 | 1.74 | 1.7e-03 |
| Lbx2\_3869 |  | mls-2 | 93 | 1.29 | 1.7e-03 |
| V$HOX13\_01 |  | lin-39 | 63 | 1.56 | 1.7e-03 |
| MA0227.1 |  | ceh-32 lin-39 | 92 | 1.30 | 1.7e-03 |
| HMGA1\_f1 |  | let-381 mel-28 Y116A8C.22 | 84 | 1.36 | 1.8e-03 |
| UNCX\_1 |  | alr-1 (0.67) cfi-1 ceh-14 | 41 | 1.92 | 1.8e-03 |
| MA0071.1 |  | nhr-6 (0.54) nhr-213 nhr-71 nhr-68 | 46 | 1.80 | 1.9e-03 |
| BARHL2\_4 |  | ceh-31 | 39 | 1.96 | 1.9e-03 |
| MA0083.2 |  | unc-120 | 50 | 1.73 | 2.0e-03 |
| pTH5778 |  | egl-5 | 39 | 1.95 | 2.0e-03 |
| SOX10\_1 |  | egl-13 (0.61) sox-4 | 71 | 1.47 | 2.1e-03 |
| Lhx1\_2240 |  | lim-7 | 38 | 1.98 | 2.1e-03 |
| Hoxa5\_3415 |  | lin-39 | 39 | 1.95 | 2.2e-03 |
| Hoxa2\_3079 |  | lin-39 | 39 | 1.94 | 2.2e-03 |
| pTH9059 |  | ztf-28 che-1 | 41 | 1.89 | 2.3e-03 |
| pTH10630 |  | lsy-27 | 42 | 1.87 | 2.4e-03 |
| Etv6 |  | lin-1 C24A1.2 | 52 | 1.68 | 2.5e-03 |
| pnr\_SANGER\_5\_FBgn0003117 |  | elt-1 | 65 | 1.52 | 2.5e-03 |
| Meox1\_2310 |  | ceh-31 | 42 | 1.86 | 2.5e-03 |
| pTH9958 |  | ztf-6 | 82 | 1.36 | 2.6e-03 |
| I$E74A\_01 |  | lin-1 C24A1.2 | 17 | 3.25 | 2.6e-03 |
| pTH9137 |  | nhr-65 | 58 | 1.59 | 2.7e-03 |
| pTH9250 |  | C34D1.1 | 57 | 1.61 | 2.8e-03 |
| pTH5812 |  | ceh-14 | 36 | 2.00 | 2.9e-03 |
| Hoxb5\_3122 |  | lin-39 | 38 | 1.93 | 3.1e-03 |
| CG14962\_SANGER\_5\_FBgn0035407 |  | C34H4.5 | 79 | 1.38 | 3.3e-03 |
| POU2F2\_2 |  | unc-86 ceh-18 | 61 | 1.55 | 3.3e-03 |
| Hmbox1\_2674 |  | hmbx-1 | 32 | 2.09 | 3.8e-03 |
| pTH8991 |  | cey-3 | 58 | 1.57 | 4.1e-03 |
| Vsx1\_1728 |  | alr-1 (0.67) | 17 | 3.11 | 4.1e-03 |
| pTH6482 |  | ceh-19 | 20 | 2.76 | 4.2e-03 |
| PDX1\_1 |  | ceh-43 ceh-1 ceh-31 lin-39 | 35 | 1.98 | 4.3e-03 |
| PBX1\_do |  | ceh-20 lin-39 ceh-12 | 52 | 1.64 | 4.4e-03 |
| Elf5 |  | C24A1.2 | 50 | 1.67 | 4.4e-03 |
| VENTX\_1 |  | dve-1 pha-2 ceh-45 ceh-53 | 40 | 1.85 | 4.5e-03 |
| Mv129 |  | ceh-18 sox-4 tbp-1 | 76 | 1.39 | 4.6e-03 |
| TFE3\_f1 |  | hlh-30 pax-1 | 101 | 1.21 | 4.6e-03 |
| MEF2A\_1 |  | mef-2 | 76 | 1.39 | 4.8e-03 |
| pTH9913 |  | skn-1 | 61 | 1.53 | 4.8e-03 |
| V$TBP\_01 |  | tbp-1 | 68 | 1.46 | 5.0e-03 |
| Hoxb3\_1720 |  | lin-39 | 38 | 1.88 | 5.2e-03 |
| pTH9082 |  | mab-23 | 83 | 1.33 | 5.3e-03 |
| pTH10645 |  | nhr-7 | 66 | 1.47 | 5.3e-03 |
| pTH5922 |  | ceh-24 | 34 | 1.97 | 6.0e-03 |
| Gm4881 |  | F19F10.1 lin-1 | 43 | 1.76 | 6.3e-03 |
| EMX1\_2 |  | ceh-2 ceh-16 | 67 | 1.45 | 6.4e-03 |
| pTH6447 |  | ceh-19 | 12 | 3.95 | 6.5e-03 |
| Sox17\_2837 |  | sox-4 | 68 | 1.44 | 6.6e-03 |
| SPDEF\_2 |  | lin-1 | 55 | 1.57 | 6.6e-03 |
| Hoxb4\_2627 |  | lin-39 | 36 | 1.90 | 6.9e-03 |
| pTH10038 |  | gei-3 | 65 | 1.47 | 6.9e-03 |
| pTH9242 |  | mel-28 | 61 | 1.51 | 6.9e-03 |
| hth\_SOLEXA\_2\_FBgn0001235 |  | ceh-32 ceh-20 | 65 | 1.47 | 7.0e-03 |
| Hoxb8\_3780 |  | lin-39 | 38 | 1.85 | 7.0e-03 |
| pTH9884 |  | tbx-39 | 49 | 1.65 | 7.2e-03 |
| Barx2\_3447 |  | ceh-43 | 36 | 1.89 | 7.2e-03 |
| SPDEF\_6 |  | lin-1 | 39 | 1.82 | 7.3e-03 |
| Hoxc4\_3491 |  | lin-39 | 36 | 1.89 | 7.3e-03 |
| N$SKN1\_01 |  | ceh-2 skn-1 | 48 | 1.66 | 7.4e-03 |
| V$XFD3\_01 |  | cfi-1 let-381 | 71 | 1.41 | 7.4e-03 |
| pTH3751 |  | tbx-39 | 62 | 1.49 | 7.5e-03 |
| Tin\_SOLEXA\_FBgn0004110 |  | ceh-24 dsc-1 ceh-22 | 39 | 1.82 | 7.6e-03 |
| Barx1\_2877 |  | ceh-43 | 25 | 2.28 | 7.7e-03 |
| TBX2\_f1 |  | tbx-39 | 63 | 1.48 | 7.7e-03 |
| pTH10816 |  | dmd-6 | 57 | 1.54 | 7.9e-03 |
| V$FREAC2\_01 |  | fkh-8 lin-31 let-381 daf-16 fkh-7 | 45 | 1.70 | 8.0e-03 |
| rn\_SOLEXA\_5\_FBgn0259172 |  | lin-29 | 80 | 1.34 | 8.0e-03 |
| Hoxa6\_1040 |  | lin-39 | 29 | 2.09 | 8.4e-03 |
| I$UBX\_01 |  | lin-39 | 40 | 1.79 | 8.5e-03 |
| pTH9928 |  | jun-1 | 59 | 1.51 | 8.8e-03 |
| Elf3 |  | C24A1.2 | 50 | 1.61 | 9.5e-03 |
| V$TCF11\_01 |  | skn-1 | 63 | 1.47 | 9.7e-03 |
| NEUROG2\_1 |  | ngn-1 | 53 | 1.57 | 1.0e-02 |
| MA0452.2 |  | B0310.2 | 62 | 1.47 | 1.0e-02 |
| ONEC2\_si |  | ceh-48 | 106 | 1.15 | 1.0e-02 |
| pTH5561 |  | nhr-239 | 96 | 1.22 | 1.0e-02 |
| pTH10041 |  | ztf-29 | 27 | 2.14 | 1.0e-02 |
| V$CEBP\_01 |  | C48E7.11 | 61 | 1.48 | 1.0e-02 |
| pTH5714 |  | nhr-239 | 43 | 1.71 | 1.1e-02 |
| pTH3998 |  | tbx-39 | 61 | 1.48 | 1.1e-02 |
| Sox1\_2631 |  | sox-4 | 64 | 1.45 | 1.1e-02 |
| pTH9246 |  | lin-31 let-381 fkh-10 C34D1.1 | 77 | 1.35 | 1.1e-02 |
| Tcf3\_3787 |  | pop-1 | 67 | 1.42 | 1.2e-02 |
| pTH9080 |  | mnm-2 | 28 | 2.07 | 1.2e-02 |
| TBX1\_1 |  | tbx-39 mab-9 tbx-43 | 27 | 2.11 | 1.2e-02 |
| pTH9901 |  | ceh-24 php-3 D1005.3 | 43 | 1.70 | 1.2e-02 |
| pTH9244 |  | tbx-39 | 36 | 1.83 | 1.2e-02 |
| pTH9182 |  | tbx-39 | 58 | 1.50 | 1.3e-02 |
| pTH10650 |  | nhr-153 | 53 | 1.55 | 1.3e-02 |
| TCF7L1\_1 |  | nhr-100 pop-1 | 67 | 1.42 | 1.3e-02 |
| H1-hESC\_RFX5\_Stanford |  | daf-19 | 57 | 1.51 | 1.3e-02 |
| Hoxa7\_2668 |  | lin-39 | 37 | 1.81 | 1.3e-02 |
| pTH9054 |  | npax-1 | 60 | 1.48 | 1.3e-02 |
| Hoxa4\_3426 |  | lin-39 | 37 | 1.80 | 1.3e-02 |
| crp\_SANGER\_10\_FBgn0001994 |  | hlh-11 | 60 | 1.48 | 1.3e-02 |
| NR1D1\_f1 |  | nhr-118 | 106 | 1.15 | 1.3e-02 |
| IRX2\_1 |  | irx-1 | 65 | 1.43 | 1.3e-02 |
| NR2F6\_f1 |  | nhr-2 | 49 | 1.60 | 1.4e-02 |
| V$HLF\_01 |  | ces-2 | 12 | 3.56 | 1.4e-02 |
| sqz\_SANGER\_5\_FBgn0010768 |  | lin-29 fkh-7 | 82 | 1.30 | 1.5e-02 |
| pTH9925 |  | ztf-11 | 78 | 1.33 | 1.5e-02 |
| pTH9245 |  | ceh-18 | 58 | 1.49 | 1.5e-02 |
| I$DFD\_01 |  | lin-39 | 54 | 1.53 | 1.5e-02 |
| PAX5\_f1 |  | pax-3 (-0.61) pax-2 | 86 | 1.27 | 1.5e-02 |
| PO3F2\_si |  | ceh-18 | 67 | 1.41 | 1.6e-02 |
| pTH9247 |  | dmd-3 C34D1.1 | 54 | 1.53 | 1.6e-02 |
| Eip74EF\_FlyReg\_FBgn0000567 |  | unc-120 C24A1.2 | 71 | 1.37 | 1.6e-02 |
| Elf4 |  | C24A1.2 | 47 | 1.61 | 1.6e-02 |
| HSFY2\_1 |  | mab-3 hsf-1 | 59 | 1.47 | 1.6e-02 |
| disco-r-Cl1\_SANGER\_5\_FBgn0042650 |  | F55C5.11 | 43 | 1.66 | 1.7e-02 |
| Foxk1\_1 |  | lin-31 nhr-213 | 38 | 1.75 | 1.7e-02 |
| Mv104 |  | nhr-2 | 95 | 1.21 | 1.8e-02 |
| pTH9216 |  | ceh-18 | 35 | 1.81 | 1.8e-02 |
| pTH9353 |  | ceh-51 (-0.52) | 56 | 1.50 | 1.8e-02 |
| twi\_da\_SANGER\_5\_FBgn0003900 |  | hlh-8 | 52 | 1.54 | 1.8e-02 |
| Nkx6-3\_3446 |  | cog-1 | 22 | 2.27 | 1.9e-02 |
| Pou3f4\_3773 |  | ceh-6 | 64 | 1.42 | 1.9e-02 |
| MA0124.1 |  | ceh-24 | 32 | 1.88 | 1.9e-02 |
| Sox21\_3417 |  | sox-4 | 62 | 1.44 | 1.9e-02 |
| pTH5914 |  | attf-1 | 74 | 1.34 | 2.0e-02 |
| SP1\_f1 |  | klf-2 | 57 | 1.48 | 2.0e-02 |
| ZNF75A\_1 |  | lag-1 ztf-3 | 47 | 1.59 | 2.0e-02 |
| Hoxa7\_3750 |  | lin-39 | 58 | 1.47 | 2.1e-02 |
| pTH6486 |  | nhr-145 | 46 | 1.60 | 2.1e-02 |
| GM12878\_ETS1\_HudsonAlpha |  | nhr-273 lin-1 | 60 | 1.45 | 2.1e-02 |
| pTH3712 |  | D1081.8 | 49 | 1.56 | 2.1e-02 |
| pTH9254 |  | mel-28 | 57 | 1.47 | 2.2e-02 |
| MA0126.1 |  | lin-48 (0.65) pax-3 (-0.61) | 51 | 1.54 | 2.2e-02 |
| Bbx\_3753 |  | lim-6 gei-3 | 25 | 2.09 | 2.2e-02 |
| pTH3064 |  | crh-1 | 42 | 1.65 | 2.3e-02 |
| CDC5L\_si |  | hlh-32 D1081.8 | 65 | 1.40 | 2.3e-02 |
| pTH10807 |  | F13H6.1 (0.78) | 17 | 2.58 | 2.4e-02 |
| pTH5924 |  | nhr-255 (0.65) | 40 | 1.68 | 2.4e-02 |
| pTH10805 |  | ztf-16 | 48 | 1.56 | 2.4e-02 |
| Nkx6-1\_2825 |  | cog-1 | 22 | 2.21 | 2.5e-02 |
| MA0467.1 |  | ceh-45 | 36 | 1.75 | 2.5e-02 |
| pTH6636 |  | egl-5 | 59 | 1.45 | 2.5e-02 |
| pTH9211 |  | C01B12.2 | 20 | 2.33 | 2.5e-02 |
| MCR\_f1 |  | nhr-255 (0.65) | 55 | 1.48 | 2.6e-02 |
| V$CREB\_Q4 |  | crh-1 | 52 | 1.51 | 2.6e-02 |
| pTH6641 |  | lin-31 | 43 | 1.62 | 2.6e-02 |
| pTH5083 |  | fos-1 | 61 | 1.42 | 2.7e-02 |
| ZN384\_f1 |  | lin-29 K11D2.4 | 84 | 1.26 | 2.8e-02 |
| pTH3046 |  | Y116A8C.22 | 36 | 1.74 | 2.8e-02 |
| pTH2846 |  | lin-31 | 41 | 1.65 | 2.9e-02 |
| pTH5169 |  | cfi-1 | 52 | 1.50 | 2.9e-02 |
| pTH9335 |  | mel-28 | 45 | 1.59 | 3.0e-02 |
| Etv3 |  | lin-1 | 35 | 1.75 | 3.0e-02 |
| GRHL1\_2 |  | grh-1 | 32 | 1.82 | 3.0e-02 |
| V$LYF1\_01 |  | F26F4.8 | 61 | 1.42 | 3.1e-02 |
| CG31670\_SOLEXA\_5\_FBgn0031375 |  | CELE\_Y38H8A.5 | 35 | 1.74 | 3.2e-02 |
| HXD9\_f1 |  | lin-39 | 68 | 1.36 | 3.2e-02 |
| pTH10013 |  | nhr-168 | 66 | 1.37 | 3.2e-02 |
| pTH3037 |  | hlh-15 | 89 | 1.23 | 3.3e-02 |
| pTH5018 |  | fos-1 | 58 | 1.43 | 3.4e-02 |
| pTH9951 |  | mex-6 | 40 | 1.64 | 3.5e-02 |
| pTH10777 |  | dmd-3 | 30 | 1.85 | 3.5e-02 |
| pTH10030 |  | xbp-1 | 33 | 1.77 | 3.6e-02 |
| pTH9188 |  | dmd-5 | 30 | 1.84 | 3.6e-02 |
| pTH9924 |  | nhr-46 | 49 | 1.52 | 3.6e-02 |
| FOXO1\_si |  | daf-16 | 83 | 1.26 | 3.6e-02 |
| pTH10798 |  | Y75B8A.6 | 91 | 1.21 | 3.8e-02 |
| MA0027.1 |  | ceh-16 | 56 | 1.44 | 3.9e-02 |
| pTH10714 |  | nhr-142 | 59 | 1.42 | 3.9e-02 |
| MA0102.3 |  | C48E7.11 | 61 | 1.40 | 4.0e-02 |
| pTH9915 |  | zip-3 | 52 | 1.48 | 4.0e-02 |
| pTH9306 |  | lsl-1 | 65 | 1.37 | 4.0e-02 |
| PRRX1\_f1 |  | alr-1 (0.67) | 88 | 1.23 | 4.1e-02 |
| pTH6556 |  | lim-6 | 10 | 3.48 | 4.1e-02 |
| NR4A2\_1 |  | nhr-6 (0.54) | 24 | 2.01 | 4.3e-02 |
| V$IK2\_01 |  | F26F4.8 | 38 | 1.65 | 4.3e-02 |
| pTH9076 |  | C01G12.1 | 30 | 1.81 | 4.4e-02 |
| pTH6071 |  | C33G8.2 | 36 | 1.68 | 4.4e-02 |
| pTH9220 |  | mbr-1 | 92 | 1.20 | 4.4e-02 |
| pTH9198 |  | dmd-3 | 57 | 1.43 | 4.5e-02 |
| pTH5098 |  | F45H11.6 | 38 | 1.64 | 4.6e-02 |
| Nkx3-1\_2923 |  | ceh-24 | 35 | 1.70 | 4.6e-02 |
| Six3\_1732 |  | ceh-34 | 83 | 1.25 | 4.9e-02 |
| Hoxc13\_3127 |  | pal-1 | 58 | 1.41 | 4.9e-02 |

### Correlated (and anti-correlated) transcription factors

|  |  |
| --- | --- |
| **Transcription factor** | **Correlation** |
| ets-5 | 0.93 |
| elt-6 | 0.80 |
| nhr-181 | 0.79 |
| F13H6.1 | 0.78 |
| bed-3 | 0.77 |
| ceh-8 | 0.76 |
| nhr-216 | 0.74 |
| nhr-214 | 0.71 |
| lfi-1 | 0.68 |
| alr-1 | 0.67 |
| ZK337.2 | 0.67 |
| nhr-187 | 0.67 |
| nhr-40 | 0.67 |
| odr-7 | 0.66 |
| lin-48 | 0.65 |
| nhr-255 | 0.65 |
| npax-2 | 0.64 |
| hlh-4 | 0.63 |
| med-2 | 0.63 |
| F10B5.3 | 0.63 |
| nhr-1 | 0.63 |
| egl-13 | 0.61 |
| nhr-253 | 0.60 |
| pros-1 | 0.60 |
| nhr-188 | 0.60 |
| tbx-8 | -0.46 |
| cey-3 | -0.46 |
| nhr-2 | -0.46 |
| zip-11 | -0.47 |
| ccch-3 | -0.47 |
| spe-44 | -0.47 |
| ubxn-1 | -0.48 |
| R144.3 | -0.49 |
| ztf-28 | -0.49 |
| hmbx-1 | -0.50 |
| pqn-75 | -0.50 |
| tbx-9 | -0.51 |
| ceh-51 | -0.52 |
| Y48G9A.11 | -0.52 |
| nhr-108 | -0.52 |
| sup-35 | -0.53 |
| nhr-246 | -0.55 |
| zip-8 | -0.60 |
| T26A5.8 | -0.61 |
| pax-3 | -0.61 |
| cep-1 | -0.64 |
| F49E8.2 | -0.64 |
| nhr-171 | -0.66 |
| cey-2 | -0.68 |
| nhr-210 | -0.71 |

### ChIP peaks enriched

none found
